# Hepatic Resection for Hepatocellular Carcinoma in the Era of Molecular-targeted Agents and Immune Checkpoint Inhibitors in Japan

**DOI:** 10.31662/jmaj.2021-0035

**Published:** 2021-07-06

**Authors:** Akinobu Taketomi

**Affiliations:** 1 Department of Gastroenterological Surgery I, Graduate School of Medicine, Hokkaido University, Sapporo, Japan

**Keywords:** borderline resectable, hepatic resection, hepatocellular carcinoma, immune checkpoint inhibitor, molecular-targeted agent

## Abstract

Hepatic resection or liver transplantation for hepatocellular carcinoma (HCC) represents the only chance for achieving a cure. For the past several decades in Japan, aggressive hepatic resection has been performed for advanced HCC, with consequent good outcomes. According to the 21st Nationwide Follow-Up Survey of Primary Liver Cancer in Japan, 38.3% of patients were treated with hepatic resection or liver transplantation as the initial treatment. The median overall survival of patients who underwent surgery was 57.0 months, and the 5- and 10-year survival rates were 48.4% and 25.2%, respectively. Since 1964, a total of 10,038 liver transplants (595 deceased-donor and 9,443 living-donor transplants) have been performed in Japan. Neoplastic disease, including HCC, was reported to be the third-most common cause of liver transplantation, and the cumulative 1-, 3-, 5-, and 10-year survival rates of living-donor liver transplants for HCC were 85.0%, 76.2%, 70.9%, and 63.1%, respectively. However, molecular-targeted agents, including sorafenib and lenvatinib, have recently been developed. Furthermore, a significantly longer survival with atezolizumab, which is an immune checkpoint inhibitor, plus bevacizumab was observed compared with sorafenib for unresectable HCC patients. Herein, we review the current status of hepatic resection and liver transplantation for HCC in Japan and discuss the role of hepatic resection in the era of molecular-targeted agents and immune checkpoint inhibitors, as well as the need for a definition of borderline resectable-HCC.

## 1_Introduction

Since the early 1970s, hepatic resection for hepatocellular carcinoma (HCC) has been performed in Japan. Initially, the mortality rate following hepatic resection was relatively high, and the main cause of death was liver failure associated with intraoperative massive bleeding or small volume of remnant liver. Tsuzuki et al. reported an operative mortality of 7.2% and 3-year survival rate of only 31% among patients with HCC following hepatic resection in 1984 ^(1)^. However, over the years, hepatic resection has become a safe procedure, thanks to the development of surgical devices, such as the Cavitron ultrasonic surgical aspirator, which is used to reduce hemorrhaging during hepatic resection, as well as imaging methods, such as ultrasound sonography, computed tomography (CT), or magnetic resonance imaging, for the accurate prediction of the future remnant liver volume or vascular anatomy. Mortality zero series of hepatic resection have been reported from high-volume centers around the world ^(2)^.

Although anticancer agent treatment, including the internal use of 5-fluorouracil, has been attempted for HCC, drugs with a curative effect have remained elusive^(3)^. However, since the efficacy of molecular-targeted agents, such as sorafenib, for prolonging the survival of patients with advanced HCC was reported in 2008, numerous agents including immune checkpoint inhibitors have been tested ^(4), (5)^.

The role of hepatic resection in the era of molecular-targeted agents and immune checkpoint inhibitors in Japan will be discussed.

## 2_Guidelines for HCC Treatment in Japan

The Japan Society of Hepatology published the “the Guidelines for HCC treatment in Japan,” which include an algorithm for the determination of the HCC treatment strategy ^(6)^. The algorithm for treating HCC was developed based on three factors: the degree of liver damage, tumor number, and tumor size in patients with liver damage severity categorized into classes A or B. In cases with only one tumor, hepatic resection is recommended, irrespective of the tumor diameter. In cases with 2 or 3 tumors < 3 cm in size each, hepatic resection or ablation is recommended. In cases with 2 or 3 tumors ≥ 3 cm or more in size, hepatic resection or hepatic artery embolization is recommended.

Patients with major vascular invasion are also candidates for hepatic resection. For those whose liver damage is class A with vascular invasion, hepatic resection may be chosen, whereas for those with extrahepatic metastasis, chemotherapy may be selected. With regard to liver transplantation for HCC, the Milan criteria (MC) have been used as a golden standard to select candidates ^(7)^. As many patients with HCC do not meet the MC, expanded criteria were awaited. The “5-5-500” expanded criteria for HCC were proposed based on the analysis of Japanese national data, which was accepted by the public health insurance system in addition to the MC in Japan ^(8)^. Liver transplantation covered by public insurance is expected to be performed for more patients with advanced HCC than ever before under the new expanded criteria.

## 3_Hepatic Resection and Liver Transplantation for HCC in Japan

Among the patients registered in the 21st Nationwide Follow-Up Survey of Primary Liver Cancer in Japan, HCC was present in 91.4%, intrahepatic cholangiocarcinoma (ICC) in 6.0%, and combined HCC and ICC in 1.0% ^(9)^. Surgery including hepatic resection or liver transplantation was the most employed initial treatment method in 38.8%, followed by local ablation therapy (22.8%), transarterial chemoembolization (TACE) (25.3%), hepatic arterial infusion chemotherapy (3.9%), and systemic chemotherapy (1.9%). Among surgically treated HCC patients, 7,823 underwent hepatic resection, and 42 underwent liver transplantation during the period. The rate of solitary tumor was 78.6%, and the rate of portal vein invasion was 16.6%, the hepatic veins 7.7%, and the bile duct 2.8% of patients treated with hepatic resection. Hr0 (limited resection), HrS (1 subsegmentectomy), Hr1 (1 segmentectomy), Hr2 (2 segmentectomy), and Hr3 (3 segmentectomy) were performed in 29.2%, 22.0%, 24.9%, 21.6%, and 1.8% of patients, respectively. The TNM classification was stage I II/III/IV-A/IV-B in 17.8%, 49.0%, 25.3%, 6.8%, and 1.1% of patients, respectively. Among the patients with HCC who underwent hepatic resection registered between 2000 and 2011 (n = 97,536), the median overall survival (OS) was 57.0 months, and the 5- and 10-year survival rates were 48.4% and 25.2%, respectively ^(9)^.

Hepatic resection has been recognized as safe and valid following the findings of various clinical studies in Japan. Fukami et al. reported the survival of patients with multiple HCC who underwent hepatic resection compared with TACE using Japanese nationwide survey data ^(10)^. After propensity score matching, the OS at 5 years following hepatic resection was 60.0%, which was higher than that after the TACE (41.6%). Among patients with tumors ≥ 30 mm in size, the survival rate of the hepatic resection group (53.0%) was higher than that of the TACE group at 5 years (32.7%). Hidaka et al. reported a study of the good results of anatomical hepatic resection for microscopic portal invasion ^(11)^. Kaibori et al. demonstrated the safety and validity of hepatic resection for patients ≥ 75 years old with early-stage HCC ^(12)^. Among Child-Pugh A patient alone, the median OS following hepatic resection was 95.0 months, whereas that after local ablation therapy was 79.9 months, and that after TACE was 45.3 months ^(9)^. While there were differences in tumor factors and liver function among these initial treatment groups, we can see why hepatic resection is often selected as the first-line treatment option in Japan.

As for liver transplantation, from 1964 to 2019, a total of 10,038 liver transplants (595 deceased-donor and 9,443 living-donor transplants) were performed in 69 institutions in Japan ^(13)^. Among the living-donor transplants, neoplastic disease (including HCC) was detected in 20.6% of transplants, cholestatic diseases in 40.4%, hepatocellular diseases in 21.0%, acute liver failure in 9.4%, and metabolic diseases in 6.1%. The 1-, 3-, 5-, and 10-year cumulative survival rates for living-donor liver transplants for HCC were 85.0%, 76.2%, 70.9%, and 63.1%, respectively.

## 4_Ocurrence and Prevention of Postoperative Complications after Hepatic Resection in Japan

Recently, an analysis of the National Clinical Database (NCD) of Japan was conducted for several surgical procedures, which contributed to the clarification of new clinical standards and risk models ^(14), (15)^. According to this analysis, there were 53,932 patients who underwent >1 sectionectomy of the liver, except for left lateral sectionectomy, between 2011 and 2017 ^(14)^. The operative 30-day mortality rates for these procedures were 1.3%-2.1%, and the postoperative 90-day mortality rates were 2.2%-4.1%. Yokoo et al. reported the postoperative complications following hepatic resection based on NCD; they found that the rates of 90-day in-hospital mortality and overall morbidity were 3.7% and 25.7%, respectively, and those of surgical site infection and bile leakage were 9.0% and 8.0%, respectively ^(16)^. Yamashita et al. also reported that bile leakage occurred in 726 patients (7.2%) among the 10,102 registered patients who underwent hepatic resection for HCC. The risk factors for bile leakage were male sex, diabetes mellitus, low hemoglobin, low albumin, central bisectionectomy, left trisectionectomy, right anterior sectionectomy, and S5 or S8 segmentectomy ^(15)^.

The most serious postoperative complication is post-hepatectomy liver failure (PHLF), which is caused by a small remnant liver volume, excessive hemorrhaging during surgery, or severe complications after hepatic resection. PHLF is a fatal condition that leads to death after hepatic resection ^(17)^. Although the rate of PHLF occurrence has been recently decreased, preventative efforts are still needed. Portal vein embolization (PVE) is a popular and safe method for increasing the remnant liver volume after hepatic resection. PVE, which induces hypertrophy of the future liver remnant (FLR), is widely used for major liver resection ^(18), (19)^. At our institution, preoperative PVE is routinely performed on patients with a remnant liver rate <40%. To calculate whether or not the estimated FLR was sufficient, 3D-CT and ^99m^Tc-galactosyl-human serum albumin (^99m^Tc-GSA) scintigraphy before and after PVE were examined ^(19)^. Sakuhara et al. reported that the mean increase in the ratio of FLR following PVE was 33.6%, and the mean ratio of FLR to the total estimated liver volume increased by 10% ^(20)^. The performance rate of planned surgery is about ≥ 80% after PVE ^(20)^. Contrarily, one-third of HCC patients were reportedly unable to undergo subsequent hepatic resection after PVE due to an insufficient FLR volume in 40% and disease progression in 33% ^(21)^. Tsuruga et al. reported that both the volume and function after PVE need to be measured to determine the optimal timing and surgical method of hepatic resection because of the functional transition lagging behind the increase in FLR ^(19)^. ^99m^Tc-GSA combined with CT volumetry or gadoxetic acid-enhanced magnetic resonance imaging is useful for predicting the FLR ^(19), (22)^. Although the postoperative outcome after hepatic resection has been improved through various means, the rate of postoperative mortality or morbidity remains high. Further efforts will be needed to improve the safety of perioperative management after hepatic resection.

## 5_Molecular-targeted Agents and Immune Checkpoint Inhibitors for HCC

In Japan, sorafenib and lenvatinib have been approved by the public health insurance system as first-line molecular-targeted agents for unresectable, advanced HCC, and regorafenib (only for cases that can tolerate sorafenib), cabozantinib, and ramucirumab (only for cases with an AFP ≥ 400 ng/mL) have been approved as second-line drugs (Table 1) ^(4), (23), (24), (25), (26), (27)^. The phase 3 trial exerted a significant effect on the OS with sorafenib (10.7 months) vs. placebo (7.9 months) ^(4)^. Lenvatinib monotherapy is recognized as the first-line treatment for unresectable HCC according to the phase 3 REFLECT study, which demonstrated that it is statistically as effective as sorafenib for improving the OS (13.6 vs. 12.3 months, respectively) ^(24)^.

**Table 1.** Summary of Phase III Clinical Trials of Molecular Targeting Agents and Immune Checkpoint Inhibitors for HCC.

No.	Year	Trial	line	Design	median OS (mo)	HR (95%CI)	Ref no.
1	2008	SHARP	1st	Sorafenib	10.7	0.69 (0.55-0.87)	^(4)^
				placebo	7.9		
2	2009	Asian-Pacific	1st	Sorafenib	6.5	0.68 (0.50-0.93)	^(23)^
				placebo	4.2		
3	2018	REFLECT	1st	Lenvatinib	13.6	0.92 (0.79-1.06)	^(24)^
				Sorafenib	12.3		
4	2017	RESORCE	2nd	Regorafenib	10.6	0.63 (0.50-0.79)	^(25)^
				Placebo	7.8		
5	2018	CELESTIAL	2nd	Cabozantinib	10.2	0.76 (0.63-0.92)	^(26)^
				Placebo	8.0		
6	2019	REACH-2	2nd	Ramucirumab	8.5	0.71 (0.53-0.95)	^(27)^
				Placebo	7.3		
7	2020	IMbrave150	1st	Atezolizumab + Bevacizumab	19.2	0.66 (0.52-0.85)	^(28)^
				Sorafenib	13.4		

HR, hazard ratio; OS, overall survival; Ref., reference.

Furthermore, in 2020, the FDA and Japanese government approved atezolizumab, which is an immune checkpoint inhibitor, in combination with bevacizumab for patients with unresectable locally advanced or metastatic HCC with no prior systemic treatment. The approval was based on the findings of a phase 3 study, which randomly allocated patients to either atezolizumab plus bevacizumab treatment or sorafenib treatment ^(28)^. The estimated median OS was 19.2 months in the atezolizumab-bevacizumab group and 13.2 months in the sorafenib group (hazard ratio, 0.58; 95% confidence interval, 0.42-0.79).

## 6_Borderline Resectable-HCC

For the past several decades, aggressive hepatic resection for advanced HCC has been performed to achieve R0 resection in Japan. Of the 891 HCC patients who underwent hepatic resection at our hospital, 13 (1.5%) were diagnosed with advanced HCC with tumor thrombus in the inferior vena cava or right atrium that was removed *via* hepatic vascular exclusion and/or cardiopulmonary bypass ^(29)^. The median OS was 15.3 months for all patients and 30.8 months for patients who underwent curative surgical resection ^(29)^. Furthermore, a large cohort study was conducted to examine the validity of hepatic resection for HCC with tumor thrombus in the main portal vein (Vp3 or Vp4). It was found that the median OS was 18.7 months ^(30)^. However, these aggressive surgical therapies might be accepted as therapeutic options as the outcomes of other therapeutic modalities are relatively low compared with those with aggressive surgery. As aforementioned, in this new era with a median OS of 19.2 months in patients treated with atezolizumab-bevacizumab, surgeons should select the optimal therapy for those with locally advanced HCC even if the case is technically resectable.

The term “borderline resectable” pancreatic adenocarcinoma (BR-PDCA) refers to a disease with major venous involvement and/or arterial abutment on thin-slice cross-sectional imaging ^(31)^. Based on this concept of categorization, numerous rigorous clinical trials of BR-PDCA have been designed ^(32), (33)^. This term should also be adopted in the field of HCC as borderline resectable-HCC (BR-HCC). To define BR-HCC, several factors should be considered, including tumor size, tumor number, macroscopic portal invasion, arterial invasion, and venous invasion. Comorbid criteria, such as the Milan criteria, up-to-seven criteria, Barcelona Clinic Liver Cancer staging, or Cancer of the Liver Italian Program score, might also be good indicators of BR-HCC. Once the definition of BR-HCC has been established, a structured clinical study should be conducted for BR-HCC to improve the outcomes of intermediate/advanced stage of HCC. As surgical resection for HCC represents the only chance for a cure, advancements in neo-adjuvant or adjuvant chemotherapy with hepatic resection for advanced HCC have been expected to improve the long-term outcomes.

## 7_Conclusions

The current state of hepatic resection for HCC in Japan was reviewed, and the role of hepatic resection in the era of molecular-targeted agents and immune checkpoint inhibitors was discussed. As HCC develops based on underlying liver diseases, such as hepatitis virus infection or liver cirrhosis, multidisciplinary treatment is needed to manage such patients. Optimal treatment selection based on the tumor number, size, and location as well as the liver function or patient’s general condition is needed to ensure the best patient outcome. Surgeons should be aware of the benefits and drawbacks of various treatment approaches for HCC in addition to surgery and use this understanding to improve patient prognosis.

## Article Information

### Conflicts of Interest

AT reports grants or speaker’s fee from Bayer pharma, Chugai Pharma, Eisai, and EA Pharma, Takeda Pharma.

### Acknowledgement

I thank Prof. Brian Quinn, from Japan Medical Communication (https://www.japan-mc.co.jp/), for editing a draft of this manuscript.
